# Di-μ-chlorido-bis­{[2-(di-*tert*-butyl­phosphan­yl)biphenyl-3-yl-κ^2^ *C* ^3^,*P*]palladium(II)} dichloro­methane disolvate

**DOI:** 10.1107/S1600536812051136

**Published:** 2013-01-09

**Authors:** Cedric W. Holzapfel, Bernard Omondi

**Affiliations:** a Research Centre for Synthesis and Catalysis, Department of Chemistry, University of Johannesburg, Auckland Park Kingsway Campus, PO Box 524, Auckland Park 2006, South Africa; b School of Chemistry and Physics, University of KwaZulu-Natal, Westville Campus, Private Bag X54001, Durban 4000, South Africa

## Abstract

The asymmetric unit of the title compound, [Pd_2_Cl_2_(C_20_H_26_P)_2_]·2CH_2_Cl_2_, contains one half-mol­ecule of the palladium complex and a dichloro­methane solvent mol­ecule. In the complex, two Pd^II^ atoms are bridged by two Cl atoms, with the other two coordination sites occupied by a C atom of the biphenyl system and a P atom, resulting in a distorted square-planar coordination geometry of the Pd^II^ atom and a cyclo­metallated four-membered ring. The Pd_2_Cl_2_ unit is located about an inversion center. The planes of the rings of the biphenyl system make a dihedral angle of 66.36 (11)°.

## Related literature

For background to palladacycles, see: Beletskaya & Cheprakov (2004 ▶); Orlye & Jutland (2005 ▶); Herrmann *et al.* (2003 ▶). For their applications as catalysts for meth­oxy­carbonylation, see: Omondi *et al.* (2011 ▶); Williams *et al.* (2008 ▶). For related structures with Pd^II^ in *ortho*-position, see: Sole *et al.* (2004 ▶); Mohr *et al.* (2006 ▶); Bennett *et al.* (2010 ▶); Christmann *et al.* (2006 ▶); Garrou *et al.* (1981 ▶). 
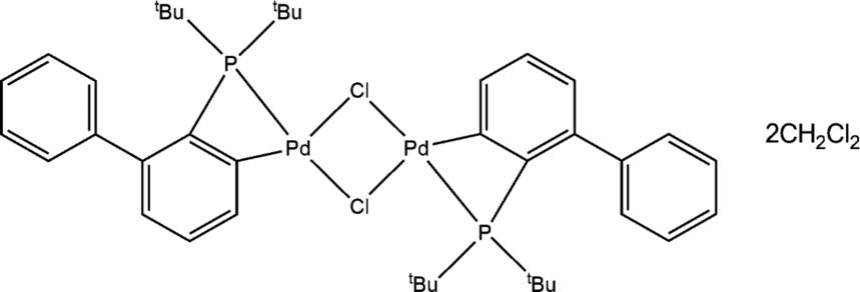


## Experimental

### 

#### Crystal data

[Pd_2_Cl_2_(C_20_H_26_P)_2_]·2CH_2_Cl_2_
*M* *_r_* = 1048.31
Triclinic, 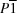

*a* = 9.4741 (15) Å
*b* = 10.1805 (15) Å
*c* = 12.0677 (18) Å
α = 103.250 (4)°
β = 95.443 (3)°
γ = 97.080 (3)°
*V* = 1115.2 (3) Å^3^
*Z* = 1
Mo *K*α radiation
μ = 1.27 mm^−1^
*T* = 100 K
0.08 × 0.07 × 0.02 mm

#### Data collection

Bruker X8 APEXII 4K KappaCCD diffractometer
Absorption correction: multi-scan (*SADABS*; Bruker, 2008 ▶) *T*_min_ = 0.905, *T*_max_ = 0.975
15104 measured reflections
5515 independent reflections
4344 reflections with *I* > 2σ(*I*)
*R*_int_ = 0.049

#### Refinement

*R*[*F* ^2^ > 2σ(*F* ^2^)] = 0.039
*wR*(*F* ^2^) = 0.094
*S* = 1.01
5515 reflections
241 parameters
1 restraint
H-atom parameters constrained
Δρ_max_ = 1.16 e Å^−3^
Δρ_min_ = −1.09 e Å^−3^

### 

Data collection: *APEX2* (Bruker, 2008 ▶); cell refinement: *SAINT-Plus* (Bruker, 2008 ▶); data reduction: *SAINT-Plus* and *XPREP* (Bruker, 2008 ▶); program(s) used to solve structure: *SHELXS97* (Sheldrick, 2008 ▶); program(s) used to refine structure: *SHELXL97* (Sheldrick, 2008 ▶); molecular graphics: *ORTEP-3* (Farrugia, 2012 ▶); software used to prepare material for publication: *WinGX* (Farrugia, 2012 ▶).

## Supplementary Material

Crystal structure: contains datablock(s) global, I. DOI: 10.1107/S1600536812051136/kj2217sup1.cif

Structure factors: contains datablock(s) I. DOI: 10.1107/S1600536812051136/kj2217Isup2.hkl

Additional supplementary materials:  crystallographic information; 3D view; checkCIF report

## Figures and Tables

**Fig. 1.**
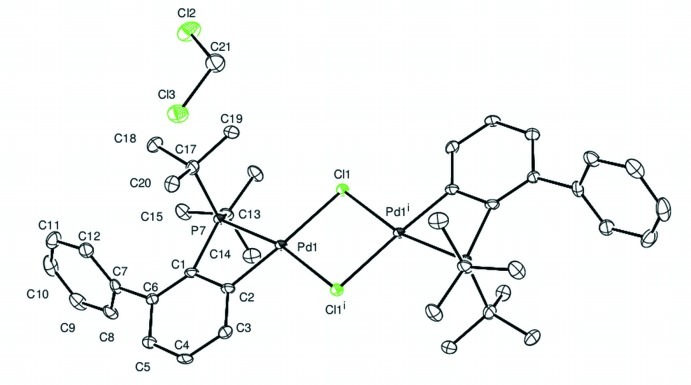
The molecular structure and atom-numbering scheme for (I), with displacement ellipsoids drawn at the 50% probability level. Hydrogen atoms have been omited for clarity. The assymetric unit only has only half a molecule of the palladium complex, the other half generated by the symetry operator -x, -y, -z.

## supplementary crystallographic information

### Comment

Palladacycles (Beletskaya & Cheprakov, 2004; Orlye & Jutland, 2005; Herrmann *et al.*, 2003) appear as long-sought thermally stable and structurally defined catalysts for Heck, cross-coupling and for methoxycarbonylation of alkenes (Omondi *et al.*, 2011, Williams *et al.*, 2008). However, with the exception of 2-(2'-di-*tert*-butylphosphine)bipheryl palladium(II) acetate, shown in the Fluka and Aldrich catalogues, this complex and other four membered ring phospha-palladacycles (Garrou, 1981) have shown little success in this regard.

The title compound crystallizes as a dichloromethane solvate, with half a molecule of the complex in the asymmetric unit, in which the the two Pd centres are bridged by two chlorides and are part of a constrained four-membered ring along with two carbon atoms of a phenyl ring and a phosphorus atom (Fig. 1). The four-membered rings are *trans* across the planar Pd_2_Cl_2_ unit, the planar Pd_2_Cl_2_ unit being on the center of inversion at 1/2, 0, 0. The coordination around the palladium atom is distorted square planar, with the angles around the metal deviating significantly from orthogonallity yet very planar. The smallest of the angles around the metal centre is 68.84 (10)° and is similar to that previously reported dinuclear cyclopalladate, 68.89 (3)° (Christmann *et al.*, 2006) and of related *ortho*-metallated Pd-structures from literature (Sole *et al.*, 2004; Mohr *et al.*, 2006; Bennett *et al.*, 2010). The other angles around the Pd centers are 85.85 (11), 95.0 (2) and 106.44 (3)° all probably due to steric hindrance caused by *tert*-butyl groups. The Pd—C distance (1.978 (3) Å) is shorter than the Pd—P distance (2.2327 (9) Å) which is in good agreement with the Pd—P separation in similar four-membered compounds with a *sp*^2^-hybridized carbon. The carbon-phosphorus distance that is part of the constrained ring is significantly shorter [1.824 (3) Å] compared to the other two [1.860 (3) and 1.879 (4) Å] as observed in most cyclopalladates. The planes of the phenyl rings of the biphenyl moiety have a dihedral angle of 66.36 (11)°.

### Experimental

(2-Biphenyl)-di-*tert*-butylphosphine (597 mg, 2 mmol) was added to the solution of lithium chloride (170 mg, 4 mmol) and palladium chloride (355 mg, 2 mmol) in 2:1 methanol-dichloromethane (15 ml) under argon. The mixture was heated under reflux under argon for 1 h. The solvent was evaporated *in vacuo*. To the residue was added dichloromethane (15 ml) and water (10 ml) and the mixture stirred for 30 minutes. The organic phase was separated, dried (anhydrous sodium sulfate) and the solvent evaporated to leave a residue (823 mg) which was chromographed over silica gel (Davisil, 20 g) using 5% methanol in dichloromethane as mobile phase. The eluent (120 ml) was evaporated to leave 1 as a colourless crystalline mass (676 mg, 82%). Good quality crystals (m.p. 162–165 °C, decomp) were obtained by diffusion of the vapours of ether into a solution of the title compound in dichloromethane at room temperature.

1H NMR (300 MHz, CDCl3): δ = 1.37 (d, *J*PH = 1.3 Hz, 36H, C(CH_3_)_3_), 7.05 (d, J = 3.2 Hz, 2H), 7.25 (dd, J = 7.5 and 3.2 Hz, 2H), 7.30–7.50 (m, 8H), 7.58 (t, J = 7.5 Hz, 2H), 7.85 (t, J = 7.5 Hz, 2H);

^13^C{^1^H} NMR (75 Mz, CDCl_3_): δ = 30.37 (d, *J*PC = 5 Hz, C(CH_3_)_3_), 35.97 (d, *J*PC = 12 Hz, C(CH_3_)_3_), 127.44 (d, *J*PC = 8.5 Hz, C-aromatic), 127.78 (s, C-aromatic), 127.44 (d, *J*PC = 8.5 Hz, C-aromatic), 127.78 (s, C-aromatic), 128.18 (s, C-aromatic), 129.19 (s, C-aromatic), 129.28 (s, C-aromatic), 133.55 (d, *J*PC = 26.0 Hz, C– aromatic), 138.55 (d, *J*PC = 32.0, C-aromatic), 141.90 (d, *J*PC = 3.9 Hz, C-aromatic), 142.81 (s, C– aromatic);

^31^P{1H} (97 MHz, CDCl_3_): δ = -17.80.

The title compound was also obtained in essentially quantitative yield by reacting the above phosphine with an equimolar amount of (*cis*, *cis*-1,5-cyclooctadiene) palladium (ll)-chloride in dichloromethane under reflux (1 hr) or at room temperature (18 hrs).

### Refinement

Carbon-bound H-atoms were placed in calculated positions [C—H = 0.98 Å for Me H atoms, 0.99 Å for Methylene H atoms and 0.95 Å for aromatic H atoms; *U*_iso_(H) = 1.2*U*_eq_(C) (1.5 for Me groups)] and were included in the refinement in the riding model approximation.

### Crystal data

**Table:**

[Pd_2_Cl_2_(C_20_H_26_P)_2_]·2CH_2_Cl_2_	*Z* = 1
*M**_r_* = 1048.31	*F*(000) = 532
Triclinic, *P*1	*D*_x_ = 1.561 Mg m^−^^3^
Hall symbol: -P 1	Mo *K*α radiation, λ = 0.71073 Å
*a* = 9.4741 (15) Å	Cell parameters from 15104 reflections
*b* = 10.1805 (15) Å	θ = 1.8–28.3°
*c* = 12.0677 (18) Å	µ = 1.27 mm^−^^1^
α = 103.250 (4)°	*T* = 100 K
β = 95.443 (3)°	Block, yellow
γ = 97.080 (3)°	0.08 × 0.07 × 0.02 mm
*V* = 1115.2 (3) Å^3^	

### Data collection

**Table:**

Bruker X8 APEXII 4K KappaCCD diffractometer	4344 reflections with *I* > 2σ(*I*)
Graphite monochromator	*R*_int_ = 0.049
φ and ω scans	θ_max_ = 28.3°, θ_min_ = 1.8°
Absorption correction: multi-scan (*SADABS*; Bruker, 2008)	*h* = −12→12
*T*_min_ = 0.905, *T*_max_ = 0.975	*k* = −13→13
15104 measured reflections	*l* = −15→16
5515 independent reflections	

### Refinement

**Table:**

Refinement on *F*^2^	Primary atom site location: structure-invariant direct methods
Least-squares matrix: full	Secondary atom site location: difference Fourier map
*R*[*F*^2^ > 2σ(*F*^2^)] = 0.039	Hydrogen site location: inferred from neighbouring sites
*wR*(*F*^2^) = 0.094	H-atom parameters constrained
*S* = 1.01	*w* = 1/[σ^2^(*F*_o_^2^) + (0.0476*P*)^2^] where *P* = (*F*_o_^2^ + 2*F*_c_^2^)/3
5515 reflections	(Δ/σ)_max_ = 0.001
241 parameters	Δρ_max_ = 1.16 e Å^−^^3^
1 restraint	Δρ_min_ = −1.09 e Å^−^^3^

### Special details

**Table:**

Geometry. All e.s.d.'s (except the e.s.d. in the dihedral angle between two l.s. planes) are estimated using the full covariance matrix. The cell e.s.d.'s are taken into account individually in the estimation of e.s.d.'s in distances, angles and torsion angles; correlations between e.s.d.'s in cell parameters are only used when they are defined by crystal symmetry. An approximate (isotropic) treatment of cell e.s.d.'s is used for estimating e.s.d.'s involving l.s. planes.
Refinement. Refinement of *F*^2^ against ALL reflections. The weighted *R*-factor *wR* and goodness of fit *S* are based on *F*^2^, conventional *R*-factors *R* are based on *F*, with *F* set to zero for negative *F*^2^. The threshold expression of *F*^2^ > σ(*F*^2^) is used only for calculating *R*-factors(gt) *etc*. and is not relevant to the choice of reflections for refinement. *R*-factors based on *F*^2^ are statistically about twice as large as those based on *F*, and *R*- factors based on ALL data will be even larger. >>> The Following ALERTS were generated <<< Format: alert-number_ALERT_alert-type_alert-level text 232_ALERT_2_C Hirshfeld Test Diff (M—*X*) Pd1 – Cl1.. 5.30 su Apllying DELU restraints does not remove this alert. 250_ALERT_2_C Large U3/U1 Ratio for Average U(i,j) Tensor ···. 2.23 Visual inspections of ellipsoids show no abnormalities. *R* = 3.8%. 911_ALERT_3_C Missing # FCF Refl Between THmin & STh/*L*= 0.600 3 912_ALERT_4_C Missing # of FCF Reflections Above STh/*L*= 0.600 62 Noted, no measures taken

### Fractional atomic coordinates and isotropic or equivalent isotropic displacement parameters (Å^2^)

**Table:**

	*x*	*y*	*z*	*U*_iso_*/*U*_eq_	
C1	0.4506 (3)	0.2051 (4)	0.3645 (3)	0.0143 (7)	
C2	0.5538 (3)	0.1684 (4)	0.2933 (3)	0.0144 (7)	
C3	0.6977 (3)	0.1820 (4)	0.3385 (3)	0.0178 (7)	
H3	0.7687	0.1578	0.2902	0.021*	
C4	0.7336 (3)	0.2319 (4)	0.4564 (3)	0.0189 (7)	
H4	0.831	0.2437	0.4889	0.023*	
C5	0.6289 (3)	0.2652 (4)	0.5278 (3)	0.0178 (7)	
H5	0.6566	0.2975	0.6082	0.021*	
C6	0.4844 (3)	0.2521 (3)	0.4840 (3)	0.0133 (7)	
C7	0.3771 (3)	0.2810 (4)	0.5662 (3)	0.0161 (7)	
C8	0.3568 (4)	0.1982 (4)	0.6430 (3)	0.0198 (8)	
H8	0.4105	0.1251	0.6417	0.024*	
C9	0.2587 (4)	0.2219 (4)	0.7209 (3)	0.0245 (8)	
H9	0.2457	0.1656	0.773	0.029*	
C10	0.1794 (4)	0.3281 (4)	0.7226 (3)	0.0288 (9)	
H10	0.1098	0.3426	0.7741	0.035*	
C11	0.2018 (4)	0.4135 (4)	0.6489 (3)	0.0259 (9)	
H11	0.1495	0.4878	0.6517	0.031*	
C12	0.3009 (4)	0.3899 (4)	0.5708 (3)	0.0201 (8)	
H12	0.3164	0.4484	0.5207	0.024*	
C13	0.1586 (3)	0.0291 (4)	0.2698 (3)	0.0161 (7)	
C14	0.2346 (4)	−0.0917 (4)	0.2839 (3)	0.0221 (8)	
H14A	0.3069	−0.0628	0.3518	0.033*	
H14B	0.2812	−0.1234	0.2156	0.033*	
H14C	0.1642	−0.1661	0.2933	0.033*	
C15	0.0822 (4)	0.0747 (4)	0.3750 (3)	0.0215 (8)	
H15A	0.0348	0.1531	0.3674	0.032*	
H15B	0.1524	0.1008	0.4443	0.032*	
H15C	0.0104	−0.0006	0.3809	0.032*	
C16	0.0503 (4)	−0.0164 (4)	0.1600 (3)	0.0210 (8)	
H16A	−0.0155	−0.097	0.1637	0.032*	
H16B	0.1018	−0.0389	0.0931	0.032*	
H16C	−0.0043	0.0577	0.1532	0.032*	
C17	0.2358 (4)	0.3161 (4)	0.2158 (3)	0.0178 (7)	
C18	0.1118 (4)	0.3638 (4)	0.2795 (3)	0.0239 (8)	
H18A	0.1377	0.3755	0.3622	0.036*	
H18B	0.0258	0.2954	0.2527	0.036*	
H18C	0.0927	0.451	0.2645	0.036*	
C19	0.1888 (4)	0.2829 (4)	0.0851 (3)	0.0217 (8)	
H19A	0.1082	0.208	0.0641	0.033*	
H19B	0.2692	0.2557	0.0442	0.033*	
H19C	0.1593	0.3638	0.0641	0.033*	
C20	0.3659 (4)	0.4295 (4)	0.2461 (3)	0.0207 (8)	
H20A	0.3418	0.5083	0.2185	0.031*	
H20B	0.4469	0.3958	0.2095	0.031*	
H20C	0.3922	0.4568	0.3295	0.031*	
C21	0.3306 (4)	0.6222 (4)	−0.0511 (3)	0.0265 (9)	
H21A	0.3451	0.6825	−0.1041	0.032*	
H21B	0.3437	0.5292	−0.0916	0.032*	
Cl1	0.32540 (8)	−0.02556 (9)	−0.05326 (7)	0.01603 (17)	
Cl2	0.15439 (10)	0.61906 (11)	−0.01468 (10)	0.0332 (2)	
Cl3	0.46013 (9)	0.68133 (10)	0.07196 (8)	0.0267 (2)	
P7	0.30128 (8)	0.16081 (9)	0.24901 (7)	0.01217 (18)	
Pd1	0.46067 (2)	0.08342 (3)	0.13497 (2)	0.01217 (8)	

### Atomic displacement parameters (Å^2^)

**Table:**

	*U*^11^	*U*^22^	*U*^33^	*U*^12^	*U*^13^	*U*^23^
C1	0.0082 (14)	0.0204 (18)	0.0149 (16)	0.0042 (13)	−0.0005 (12)	0.0051 (14)
C2	0.0097 (15)	0.0189 (17)	0.0145 (16)	0.0039 (13)	−0.0009 (12)	0.0040 (14)
C3	0.0091 (15)	0.0250 (19)	0.0186 (18)	0.0056 (14)	0.0036 (13)	0.0016 (15)
C4	0.0058 (14)	0.029 (2)	0.0196 (18)	0.0048 (14)	−0.0031 (13)	0.0029 (16)
C5	0.0126 (16)	0.0218 (18)	0.0170 (17)	0.0037 (14)	−0.0014 (13)	0.0018 (15)
C6	0.0103 (15)	0.0157 (17)	0.0134 (16)	0.0029 (13)	0.0014 (12)	0.0022 (13)
C7	0.0079 (14)	0.0224 (18)	0.0123 (16)	−0.0026 (13)	0.0005 (12)	−0.0042 (14)
C8	0.0131 (16)	0.027 (2)	0.0156 (17)	−0.0011 (14)	−0.0021 (13)	0.0022 (15)
C9	0.0169 (17)	0.037 (2)	0.0172 (18)	−0.0062 (16)	0.0018 (14)	0.0069 (17)
C10	0.0174 (18)	0.040 (2)	0.021 (2)	−0.0046 (17)	0.0088 (15)	−0.0054 (18)
C11	0.0153 (17)	0.027 (2)	0.032 (2)	0.0062 (15)	0.0051 (15)	−0.0019 (18)
C12	0.0136 (16)	0.025 (2)	0.0191 (18)	0.0023 (14)	0.0015 (14)	0.0012 (15)
C13	0.0090 (15)	0.0232 (19)	0.0148 (16)	0.0007 (13)	−0.0006 (12)	0.0036 (14)
C14	0.0157 (17)	0.0221 (19)	0.029 (2)	−0.0014 (15)	0.0012 (15)	0.0091 (16)
C15	0.0147 (16)	0.032 (2)	0.0166 (18)	−0.0025 (15)	0.0038 (13)	0.0055 (16)
C16	0.0121 (16)	0.031 (2)	0.0161 (17)	−0.0043 (15)	0.0001 (13)	0.0023 (16)
C17	0.0142 (16)	0.0217 (18)	0.0171 (17)	0.0050 (14)	0.0038 (13)	0.0017 (15)
C18	0.0174 (17)	0.029 (2)	0.026 (2)	0.0107 (16)	0.0057 (15)	0.0034 (17)
C19	0.0218 (18)	0.028 (2)	0.0159 (18)	0.0101 (16)	−0.0006 (14)	0.0053 (16)
C20	0.0211 (18)	0.0208 (19)	0.0203 (18)	0.0043 (15)	0.0045 (14)	0.0039 (15)
C21	0.0205 (18)	0.031 (2)	0.027 (2)	0.0032 (16)	0.0027 (15)	0.0062 (18)
Cl1	0.0066 (3)	0.0255 (5)	0.0139 (4)	0.0042 (3)	0.0010 (3)	−0.0001 (3)
Cl2	0.0144 (4)	0.0346 (6)	0.0501 (7)	0.0044 (4)	0.0032 (4)	0.0093 (5)
Cl3	0.0176 (4)	0.0300 (5)	0.0311 (5)	0.0015 (4)	−0.0002 (4)	0.0073 (4)
P7	0.0049 (3)	0.0191 (4)	0.0116 (4)	0.0028 (3)	0.0008 (3)	0.0014 (3)
Pd1	0.00434 (11)	0.01965 (14)	0.01151 (13)	0.00282 (9)	0.00054 (8)	0.00138 (10)

### Geometric parameters (Å, º)

**Table:**

C1—C2	1.391 (5)	C14—H14C	0.98
C1—C6	1.403 (5)	C15—H15A	0.98
C1—P7	1.824 (3)	C15—H15B	0.98
C2—C3	1.398 (4)	C15—H15C	0.98
C2—Pd1	1.978 (3)	C16—H16A	0.98
C3—C4	1.389 (5)	C16—H16B	0.98
C3—H3	0.95	C16—H16C	0.98
C4—C5	1.397 (5)	C17—C18	1.532 (5)
C4—H4	0.95	C17—C20	1.535 (5)
C5—C6	1.399 (4)	C17—C19	1.544 (5)
C5—H5	0.95	C17—P7	1.879 (4)
C6—C7	1.495 (4)	C18—H18A	0.98
C7—C12	1.389 (5)	C18—H18B	0.98
C7—C8	1.400 (5)	C18—H18C	0.98
C8—C9	1.387 (5)	C19—H19A	0.98
C8—H8	0.95	C19—H19B	0.98
C9—C10	1.388 (6)	C19—H19C	0.98
C9—H9	0.95	C20—H20A	0.98
C10—C11	1.391 (6)	C20—H20B	0.98
C10—H10	0.95	C20—H20C	0.98
C11—C12	1.396 (5)	C21—Cl2	1.764 (4)
C11—H11	0.95	C21—Cl3	1.771 (4)
C12—H12	0.95	C21—H21A	0.99
C13—C15	1.529 (5)	C21—H21B	0.99
C13—C14	1.534 (5)	Cl1—Pd1^i^	2.4154 (8)
C13—C16	1.539 (4)	Cl1—Pd1	2.4444 (9)
C13—P7	1.860 (3)	P7—Pd1	2.2328 (9)
C14—H14A	0.98	Pd1—Cl1^i^	2.4154 (8)
C14—H14B	0.98		
			
C2—C1—C6	122.1 (3)	H15A—C15—H15C	109.5
C2—C1—P7	95.0 (2)	H15B—C15—H15C	109.5
C6—C1—P7	142.8 (3)	C13—C16—H16A	109.5
C1—C2—C3	120.7 (3)	C13—C16—H16B	109.5
C1—C2—Pd1	109.9 (2)	H16A—C16—H16B	109.5
C3—C2—Pd1	129.1 (3)	C13—C16—H16C	109.5
C4—C3—C2	117.9 (3)	H16A—C16—H16C	109.5
C4—C3—H3	121	H16B—C16—H16C	109.5
C2—C3—H3	121	C18—C17—C20	110.1 (3)
C3—C4—C5	121.1 (3)	C18—C17—C19	109.4 (3)
C3—C4—H4	119.4	C20—C17—C19	108.6 (3)
C5—C4—H4	119.4	C18—C17—P7	115.1 (3)
C4—C5—C6	121.7 (3)	C20—C17—P7	106.2 (2)
C4—C5—H5	119.1	C19—C17—P7	107.3 (2)
C6—C5—H5	119.1	C17—C18—H18A	109.5
C5—C6—C1	116.4 (3)	C17—C18—H18B	109.5
C5—C6—C7	118.7 (3)	H18A—C18—H18B	109.5
C1—C6—C7	124.8 (3)	C17—C18—H18C	109.5
C12—C7—C8	119.3 (3)	H18A—C18—H18C	109.5
C12—C7—C6	121.9 (3)	H18B—C18—H18C	109.5
C8—C7—C6	118.7 (3)	C17—C19—H19A	109.5
C9—C8—C7	120.5 (4)	C17—C19—H19B	109.5
C9—C8—H8	119.8	H19A—C19—H19B	109.5
C7—C8—H8	119.8	C17—C19—H19C	109.5
C8—C9—C10	119.9 (4)	H19A—C19—H19C	109.5
C8—C9—H9	120.1	H19B—C19—H19C	109.5
C10—C9—H9	120.1	C17—C20—H20A	109.5
C9—C10—C11	120.1 (4)	C17—C20—H20B	109.5
C9—C10—H10	120	H20A—C20—H20B	109.5
C11—C10—H10	120	C17—C20—H20C	109.5
C10—C11—C12	120.0 (4)	H20A—C20—H20C	109.5
C10—C11—H11	120	H20B—C20—H20C	109.5
C12—C11—H11	120	Cl2—C21—Cl3	111.6 (2)
C7—C12—C11	120.2 (4)	Cl2—C21—H21A	109.3
C7—C12—H12	119.9	Cl3—C21—H21A	109.3
C11—C12—H12	119.9	Cl2—C21—H21B	109.3
C15—C13—C14	109.0 (3)	Cl3—C21—H21B	109.3
C15—C13—C16	110.7 (3)	H21A—C21—H21B	108
C14—C13—C16	108.7 (3)	Pd1^i^—Cl1—Pd1	92.33 (3)
C15—C13—P7	114.4 (2)	C1—P7—C13	112.74 (15)
C14—C13—P7	105.4 (2)	C1—P7—C17	112.13 (16)
C16—C13—P7	108.4 (2)	C13—P7—C17	114.90 (15)
C13—C14—H14A	109.5	C1—P7—Pd1	85.84 (11)
C13—C14—H14B	109.5	C13—P7—Pd1	115.73 (12)
H14A—C14—H14B	109.5	C17—P7—Pd1	112.17 (11)
C13—C14—H14C	109.5	C2—Pd1—P7	68.84 (10)
H14A—C14—H14C	109.5	C2—Pd1—Cl1^i^	97.07 (10)
H14B—C14—H14C	109.5	P7—Pd1—Cl1^i^	165.87 (3)
C13—C15—H15A	109.5	C2—Pd1—Cl1	174.81 (10)
C13—C15—H15B	109.5	P7—Pd1—Cl1	106.44 (3)
H15A—C15—H15B	109.5	Cl1^i^—Pd1—Cl1	87.67 (3)
C13—C15—H15C	109.5		
			
C6—C1—C2—C3	−2.4 (5)	C14—C13—P7—C1	−55.2 (3)
P7—C1—C2—C3	179.9 (3)	C16—C13—P7—C1	−171.4 (2)
C6—C1—C2—Pd1	172.0 (3)	C15—C13—P7—C17	−65.6 (3)
P7—C1—C2—Pd1	−5.8 (2)	C14—C13—P7—C17	174.7 (2)
C1—C2—C3—C4	0.5 (5)	C16—C13—P7—C17	58.4 (3)
Pd1—C2—C3—C4	−172.6 (3)	C15—C13—P7—Pd1	161.1 (2)
C2—C3—C4—C5	1.2 (6)	C14—C13—P7—Pd1	41.4 (3)
C3—C4—C5—C6	−1.2 (6)	C16—C13—P7—Pd1	−74.9 (2)
C4—C5—C6—C1	−0.6 (5)	C18—C17—P7—C1	−93.1 (3)
C4—C5—C6—C7	176.1 (3)	C20—C17—P7—C1	28.9 (3)
C2—C1—C6—C5	2.4 (5)	C19—C17—P7—C1	144.9 (2)
P7—C1—C6—C5	178.7 (3)	C18—C17—P7—C13	37.4 (3)
C2—C1—C6—C7	−174.1 (3)	C20—C17—P7—C13	159.4 (2)
P7—C1—C6—C7	2.2 (7)	C19—C17—P7—C13	−84.6 (3)
C5—C6—C7—C12	114.5 (4)	C18—C17—P7—Pd1	172.3 (2)
C1—C6—C7—C12	−69.1 (5)	C20—C17—P7—Pd1	−65.7 (2)
C5—C6—C7—C8	−63.4 (4)	C19—C17—P7—Pd1	50.3 (3)
C1—C6—C7—C8	113.0 (4)	C1—C2—Pd1—P7	5.1 (2)
C12—C7—C8—C9	1.8 (5)	C3—C2—Pd1—P7	178.8 (4)
C6—C7—C8—C9	179.8 (3)	C1—C2—Pd1—Cl1^i^	−176.0 (2)
C7—C8—C9—C10	0.3 (5)	C3—C2—Pd1—Cl1^i^	−2.3 (3)
C8—C9—C10—C11	−2.2 (6)	C1—P7—Pd1—C2	−3.63 (15)
C9—C10—C11—C12	1.9 (6)	C13—P7—Pd1—C2	−116.91 (16)
C8—C7—C12—C11	−2.0 (5)	C17—P7—Pd1—C2	108.59 (16)
C6—C7—C12—C11	−180.0 (3)	C1—P7—Pd1—Cl1^i^	−8.08 (19)
C10—C11—C12—C7	0.2 (5)	C13—P7—Pd1—Cl1^i^	−121.36 (17)
C2—C1—P7—C13	121.0 (2)	C17—P7—Pd1—Cl1^i^	104.15 (17)
C6—C1—P7—C13	−55.8 (5)	C1—P7—Pd1—Cl1	174.08 (11)
C2—C1—P7—C17	−107.4 (2)	C13—P7—Pd1—Cl1	60.80 (12)
C6—C1—P7—C17	75.7 (5)	C17—P7—Pd1—Cl1	−73.70 (12)
C2—C1—P7—Pd1	4.8 (2)	Pd1^i^—Cl1—Pd1—P7	179.47 (3)
C6—C1—P7—Pd1	−172.0 (4)	Pd1^i^—Cl1—Pd1—Cl1^i^	0
C15—C13—P7—C1	64.6 (3)		

Symmetry code: (i) −*x*+1, −*y*, −*z*.
